# Topical administration of *S. salivarius* 24SMB-*S. oralis* 89a in children with adenoidal disease: a double-blind controlled trial

**DOI:** 10.1007/s00431-023-05192-w

**Published:** 2023-10-24

**Authors:** Francesco Folino, Daniele Di Pasquale, Paola Marchisio, Lorenzo Pignataro, Pasquale Capaccio, Lorenzo Gaini, Ludovica Battilocchi, Samantha Bosis, Sara Torretta

**Affiliations:** 1 Fondazione IRCCS Cà Granda Ospedale Maggiore Policlinico di Milano, Milan, Italy; 2 Department of Pathophysiology and Transplantation, University of Milan, Milan, Italy; 3 Department of Clinical Sciences and Community Health, University of Milan, Università degli Studi di Milano, Fondazione IRCCS Cà Granda Ospedale Maggiore Policlinico, Via F. Sforza 35, 20122 Milan, Italy; 4 Department of Biomedical Surgical Dental Science, University of Milan, Milan, Italy

**Keywords:** Otitis, Adenoids, Children

## Abstract

Chronic adenoiditis (CA) is generally sustained by some infectious foci mainly located within the nasopharynx or in the deep adenoidal pads and it is characterized by a complex interplay between bacterial species. The aim of this study was to assess the efficacy and safety of the topical nasal administration of a probiotic compound based on *S. salivarius* 24SMB and *S. oralis* 89a in children with CA in terms of reduction in: the number of acute adenoidal infections (primary outcome), and in the blockage of the nasopharynx space by hypertrophic adenoids (secondary outcome). A prospective, double-blind, 1:1 randomized controlled study was performed to test the effectiveness of a 90-day treatment with Rinogermina spray (DMD ITALIA s.r.l, Rome), 1 puff each nostril twice a day for 90 days, to nasal spray placebo in children with CA (in terms of number of acute exacerbations and blockage of nasopharynx space assessed after 90 days of treatment- T1, and 90 days later- T2). The final analysis was based on 152 children (males = 48.0%; mean age = 49.2 ± 14.1 months). Compared to the baseline, no significant differences in terms of number of acute exacerbations at T1 and T2 follow-up visits were detected in both groups. After treatment, a significant reduction in the blockage of nasopharynx space by hypertrophic adenoids (0.002 < *p*-value < 0.007) compared to the baseline was attested in the study group at T1 and T2, but not in the control group.

*Conclusions*: Our findings document a positive effect of Rinogermina spray in achieving reduction in the blockage of nasopharynx space by hypertrophic adenoids, thus suggesting that its use into the integrated therapeutic management of children with CA could be of a certain utility.

**Table:** 

**What is Known:** *• Chronic adenoiditis in children results from an imablance in baterial homeostasis at the nasophaynx, with impairment in respiratory microbiota.* *• The modulatory effect of target transnasal bacteriotheray by means of S. salivarius has been considered in children with chronic adenoiditis in children with recurrent acute otitis media with preliminary positive results.*	
**What is New:** *• This randomized controlled study, specifically designed on a cohrt of children with chronic adenoiditis, documents a certain effectiveness of the probiotic treatment in achieving a reduction in the grade of adenoidal hypertropy, compared to placebo.*

**What is Known:**

*• Chronic adenoiditis in children results from an imablance in baterial homeostasis at the nasophaynx, with impairment in respiratory microbiota.*

*• The modulatory effect of target transnasal bacteriotheray by means of S. salivarius has been considered in children with chronic adenoiditis in children with recurrent acute otitis media with preliminary positive results.*

**What is New:**

*• This randomized controlled study, specifically designed on a cohrt of children with chronic adenoiditis, documents a certain effectiveness of the probiotic treatment in achieving a reduction in the grade of adenoidal hypertropy, compared to placebo.*

## Introduction

Chronic adenoiditis (CA) is a common condition in pediatric otorhinolaryngological clinical practice, frequently associated with chronic or recurrent middle ear infections, rhinosinusitis, and sleep disordered breathing complaints; these situations often require specialist consultations, antibiotic prescriptions and, in some cases, surgical treatment [1, 2].

Chronic or recurrent inflammations of the nasopharyngeal and middle ear district are generally bacterial diseases, mainly sustained by an infectious focus located within the nasopharynx or in the deep adenoidal pads; it is characterized by a complex interplay between different bacterial species, sometimes within the setting of a resistant bacterial biofilm [3–9]. In addition, some studies documented that structural organization and spatial location of multi-species bacterial community (including both saprophytes and pathogens) over human respiratory mucosa (the so-called respiratory microbiome) is crucial in determining individual predisposition to infections [10–12]. With regard to this, the role of respiratory microbiome in children with chronic adenoiditis and recurrent acute otitis media has been recently investigated with the finding of peculiar pattern of distribution and composition of multiple-species bacterial micro-community [11–15]. In addition, it has been documented that infectious recurrences are related to a shift in bacterial composition, with a more frequent detection of a certain bacterial core characterized by enhanced pathogenicity of some species and increased tolerance of other ones [13]. In the pediatric age, the more prevalent species belongs to Haemophilus spp., Neisseria spp., S. pneumonia, and some obligate anaerobes including *Fusobacterium* spp., *Prevotella* spp., and *Porphyromonas* spp. can be detected, too [13].

Based on this, current therapeutic management of children with CA should overcome the emerging issue of increasing resistance of bacterial biofilm to traditional antibiotic treatment (which is up to 10–1000 higher than the one reported for the corresponding planktonic species), and be modulated to target the individual respiratory microbiome. Under these circumstances, some studies have suggested the effectiveness of topic probiotic treatment by means of *S. salivarius* in children with recurrent acute otitis media [16–19]; this could be ascribable to the fact that this saprophyte, acting as an antagonistic to other bacteria belonging to *Streptococcus* spp., is generally depleted within the nasopharynx of children with recurrent upper airways tract infections (URTIs), with the following facilitation to pathogens overgrowth [20].

The aim of this study was to assess the efficacy and safety of the topical nasal administration of a probiotic compound based on *S. salivarius* 24SMB and *S. oralis* 89a (Rinogermina, by DMG ITALIA S.r.l, Rome) in children with CA in terms of reduction in the number of acute adenoidal infections (primary outcome), and in the blockage of nasopharynx space by hypertrophic adenoids (secondary outcome).

## Materials and methods

### Study design and setting

This prospective, double-blind, randomized controlled study was carried out at the University of Milan’s Department of Clinical Sciences and Community Health between January 2019 and January 2021. The protocol was approved BY the Ethics Committee “Milano Area 2” (protocol number AC-TAR-2018, September 10^th^, 2018), and it was conducted in accordance with the standards of Good Clinical Practice; written informed consent was obtained from the children’s parents or legal guardians.

### Study subjects

The study involved children aged 2–18 years with CA defined as adenoidal hypertrophy detected by means of nasopharyngeal flexible video-endoscopy and recurrent infectious exacerbations (i.e., ≥ 3 episodes of acute adenoiditis requiring antibiotic treatment in the former 6 months or ≥ 4 episodes in the former 12 months) in the presence of persistent mucous secretion at the adenoidal surface [1].

The exclusion criteria were the following: previous ear surgery or adenoidectomy; previous tonsillar surgery; concomitant systemic diseases; craniofacial, neuromuscular, immunological, syndromic, or defined genetic abnormalities; choanal atresia; chronic eardrum perforation; immunomodulatory treatment, vitamin D supplementation or complementary and alternative medicine assumption in the previous 30 days; acute febrile illness; upper respiratory tract infection or antibiotic therapy in the previous 14 days.

### Interventions

Upon enrolment (T0), a complete clinical history (including the number of documented acute adenoidal infections occurring in the previous 3 months) was taken, and the child’s gender and age were recorded; all the children underwent a detailed otorhinolaryngological clinical examination including nasopharyngeal flexible video-endoscopy (performed by the same trained examiner) aimed at grading the blockage of nasopharynx space by hypertrophic adenoids expressed as the percentage of choanal space occlusion.

Upon enrolment, the patients were double-blindly randomized 1:1 via a random number generator to:

- the control group, which received a placebo nasal spray, 1 puff each nostril twice a day for 90 days;
- the study group, which received the administration of Rinogermina spray (DMD ITALIA s.r.l, Rome), 1 puff each nostril twice a day for 90 days:

The compliance to the assigned treatment was evaluated by counting the number of dispensed and returned devices.

Clinical assessment was repeated at the end of the treatment (90 days after recruitment, T1), and 90 days later (90 days after treatment suspension, T2).

### Statistical analysis

The sample size was determined on the basis of the primary endpoint, which was to evaluate the impact of the topical administration of Rinogermina nasal spray on the reduction of the number of acute adenoidal infections, and was computed using published data reporting a 15% reduction of acute exacerbation in children receiving topic probiotic treatment [16, 17]. Assuming a standard deviation of 0.08, it was calculated that 76 subjects in each group would lead to an alpha value of 0.05, and a power of 80%.

The results are given as absolute numbers and percentages, or as arithmetical mean values ± standard deviation. The dichotomous outcomes were analyzed using contingency table analysis, and continuous variables using non-parametric tests.

A *p*-value of < 0.05 was considered statistically significant, and the data were analyzed using the STATA 10.0 software (StataCorp, College Station, TX, USA).

## Results

Analysis was based on the findings relating to 152 children (males = 48.0%) with a mean age = 49.2 ± 14.1 months (range = 25–96 months), including 76 children (50%) belonging to study group (males = 47.4%; mean age = 48.6 ± 13.4 months), and 76 (50%) to the control group (males = 48.7%; mean age = 49.6 ± 15.0 months). About 92% of children had been recruited and managed between January and November 2019 and the remaining ones by February 2020; then, recruitment was interrupted given the declaration of the pandemic status in Italy. The two groups were also comparable in terms of their baseline clinical characteristics, including the number of acute adenoidal infections occurring in the previous 3 months, and the blockage of nasopharynx space by hypertrophic adenoids.

No devices were returned at the follow-up visit, and no untoward effects were reported in either group.

Compared to the baseline, no significant differences in the number of acute exacerbations at T1 and T2 follow-up visits were detected in both groups (Table 1).

**Table 1:** Clinical data in the study group and in the control group

**Study group**							
**Characteristic**	**No. of URTIs**	***p-*value**		**% of blockage of nasopharynx space**	***p-*value**		
**T0**	1.4 ± 0.3	ns*		63.1 ± 16.3	0.007*		0.002^
**T1**	1.3 ± 0.3		ns**	58.6 ± 16.4		ns**	
**T2**	1.4 ± 0.2			54.2 ± 16.9			
**Control group**							
**Characteristic**	**No. of URTIs**	***p*-value**		**% of choanal obstruction**	***p*-value**		
**T0**	1.6 ± 0.4	ns*		63.9 ± 13.9	ns*		ns^
**T1**	1.5 ± 0.5		ns**	62.1 ± 13.9		ns**	
**T2**	1.3 ± 0.4			62.4 ± 14.6			

*URTIs* upper respiratory tract infections, *No* number, *n.s*. non-significant

*T0 vs T1; **T1 vs. T2; ^T0 vs T2

**Fig. 1:**
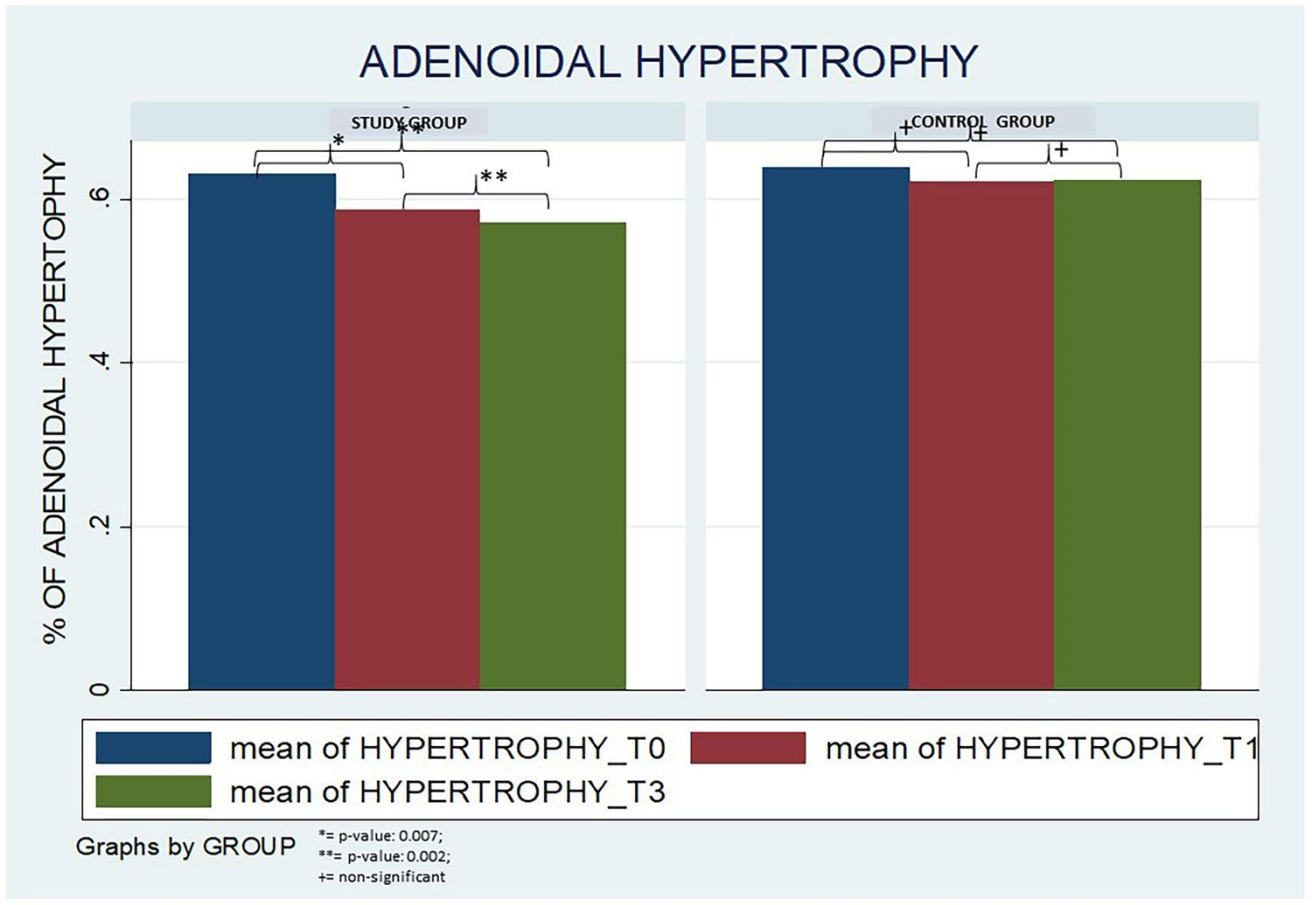
Blockage of nasopharynx space by hypertrophic adenoids in the study population (expressed as the percentage of choanal space occlusion)

With regards to the blockage of nasopharynx space by hypertrophic adenoids, a significant reduction (0.002 < *p*-value < 0.007) compared to the baseline was attested in the study group at T1 and T2, but not in the control group (Fig. 1). The corresponding figures for T0 and T2 are the following: 63.1 ± 16.3% and 54.2 ± 16.9%; 63.9 ± 13.9% and 62.4 ± 14.6%, respectively, in the study group and in the control one.

## Discussion

This study pointed to a positive effect of probiotic nasal treatment with *S. salivarius* 24SMB-*S. oralis* 89a in reducing the blockage of nasopharynx space by hypertrophic adenoids in children with CA, as we found a significant decrease in the mean percentage of choanal space occlusion by adenoidal pads in patients receiving a 3-month treatment with Rinogermina spray compared to the baseline; this positive effect was found to persist in the medium term, as it was documented also 3 months after treatment discontinuation. Otherwise, no significant differences compared to the baseline were found in the placebo group. This lines with the finding of La Mantia et al. [21] who documented a significant reduction in the number of children with large adenoidal hypertrophy after *S. salivarius* 24SMB and *S. oralis* 98a nasal spray, thus resulting in a reduced recourse to surgery. The authors assume that all the patients successfully completed the treatment protocol as no devices were returned at the follow-up visit.

The impact of topic probiotic in CA has been previously investigated by Karpova et al. [22] who performed an open randomized comparative study in 219 children with clinical and anamnestic signs of CA receiving either a 30-day treatment with *S. salivarius* K12 plus standard nasal douche therapy (113 children), or the nasal douche therapy alone (106 children), with the finding of a significant reduction in the number of exacerbation of adenoiditis both at day 30 and 90. The effect on the grade of adenoidal hypertrophy was not tested. On the contrary, our double-blind randomized controlled trial failed to find any significant impact of probiotic nasal treatment on the number of acute infections neither during the 90-day treatment period, nor in the 90-day follow-up period.

Some authors published preliminary positive results about effectiveness of nasal bacteriotherapy in children with history of recurrent acute otitis media (RAOM), a clinical condition frequently related to CA in children [16, 23]. In particular, Marchisio et al. [16] documented that RAOM-children receiving *S. salivarius* 24SMB nasal spray had a twice-fold reduced risk of experiencing any further AOM episode during the 6-month follow-up period, compared to children receiving placebo; the number of antibiotic treatments was significantly lower in the study group than in the placebo group, as well. These results line with the ones by La Mantia et al. [23] who tested the effectiveness of a combined probiotic treatment with intranasally administered *S. salivarius* 24SMB and *S. oralis* 89a as a preventive mean in RAOM, with a reported significant reduction of AOM episodes in the treatment group compared to controls, receiving no treatment.

In addition, few studies have previously investigated the role of probiotic treatment with *S. salivarius* spp. in sore-throat episodes with conflicting results [24, 25]; moreover, a systematic review recently conducted by Wilcox et al. [26] concluded that, despite safe and well-tolerated, effectiveness of *S. salivarius* K12 bacteriotherapy is to date uncertain both in the prevention and in the acute treatment of sore-throat, given the poor quality of the trials actually available, and their high risk of bias.

The potential utility of replacement bacteriotherapy as a supportive therapeutic option in the management of children with upper airway inflammations, including adenoidal disease, lines within the supposed bacterial interference resulting from the implementation of nasopharyngeal flora with harmless competitors, effectively able to exclude or counter pathogens overgrowth without significantly unbalance on the respiratory microbiome [27].

With regards to the primary outcome of the study, we did not find any significant effect of the studied treatment in terms of reduction in the number of acute adenoidal infections. This finding opposites to previous ones [28–30]. In particular, the former study by Manti et al. [28] documented the effectiveness of *S. salivarius* 24SMB- *S. oralis* 89a bacteriotherapy in reducing symptoms of airway infections (i.e., fever, cough, wheezing, otalgia, and rhinorrhoea) in 91 children with history of respiratory infections compared to the baseline, with the finding of progressive and significant therapeutic effectiveness precociously sustained in older children (i.e., those older than 3). Our results are not directly comparable, given substantial differences among the two studies which involve the study design (no control treatment in Manti’s study), criteria for patient selection (children with respiratory infections in Manti’s study vs. children with documented chronic adenoiditis in our study), and clinical outcomes. Beside these and also considering the reported enhanced therapeutic effect in older children [28], the fact that our study group included younger children (mean age 49.2 ± 14.1 months) compared to the population recruited by Manti et al. [28] (mean age 7.4 ± 2.3 years) could account for the difference.

In 2019, Passali et al. [29] described the effectiveness of prophylactic bacteriotherapy with *S. salivarius* 24SMB-*S. oralis* 89a in 141 children (mean age 7.5 years) with history of URTIs, by reporting a 63% reduction of acute infectious exacerbations compared to the baseline. They also found that the recurrence rate of patients with adenoiditis dropped from 48.2% during the first 3 months of treatment to 29.5% at 6 months, and 22.3% at the end of the follow-up (12 months). However, also in this case, no comparison against placebo has been used to control the results, so our findings resulting from a randomized controlled double-blind trial are not directly comparable to these.

The study by Bidossi et al. [30] documented the in vitro ability of *S. salivarius* 24SMB and *S. oralis* 89a to interfere with biofilm formation capability of selected respiratory pathogens, and to disperse their pre-formed biofilms, thus suggesting a possible effect as a therapeutic and preventive mean in patients with respiratory infection. Otherwise, it should be considered that the mucociliary clearance effect exerted by the respiratory epithelium could distribute and disperse the topical compound away the target site (i.e., nasopharynx), thus possibly reducing the contact time and the therapeutic effect in clinical practice.

In our study, most of the children had been recruited and managed between January and November 2019 and the remaining ones by February 2020; then, recruitment was interrupted given the declaration of the pandemic status in Italy. So we do not think that the lockdown could have influenced our results. On the contrary, some patients both in the study and in the control group had been recruited during spring or summer, and this could have partially influenced the rate of respiratory infections, given the therapeutic effect of good season. Another possible limitation of this study is related to the fact that the real clinical impact of the reduction in the blockage of the nasopharyngeal space after treatment here found to be significant should be more objectively assessed by the use of some dedicated second-level tools such as anterior rhinomanometry or polysomnography. However, their use in small children with limited adenoidal disease is not always easy to be performed in the real clinical practice, and they are not routinely recommended in the first-line diagnostic setting.

In conclusion, despite our results did not document any meaningful impact of *S. salivarius* 24SMB in reducing the number of URTIs in our cohort of children, the beneficial effect on the blockage of nasopharynx space by hypertrophic adenoids reported in the study group (but not in the placebo group) may positively impact on the overall child wee-being, with an improvement in nasal airflow patency and in sleep-disordered breathing complaints, thus reducing the burden on disease in these patients. On the other side, the impact of *S. salivarius* 24SMB-*S. oralis* 89a bacteriotherapy in the real-life setting should be more thoroughly investigated, in order to objectively quantify a possible fallout on children’s quality of life, and the stability of the results in the long-term.

## Data Availability

Data available on request due to privacy/ethical restrictions.
